# Developmentally appropriate transitional care during the Covid-19 pandemic for young people with juvenile-onset rheumatic and musculoskeletal diseases: the rationale for a position statement

**DOI:** 10.1186/s12969-021-00609-y

**Published:** 2021-08-25

**Authors:** Janet E. McDonagh, Rachel Tattersall, Jacqui Clinch, Joanne Swan, Helen E. Foster, Liza McCann

**Affiliations:** 1 Versus Arthritis Centre for Epidemiology; Centre for MSK Research, University of Manchester, Stopford Building, 2nd floor, Oxford Rd, Manchester, M13 9PT UK; 2 NIHR Biomedical Research Centre, Manchester University Hospital NHS Trust, Manchester, UK; 3 Department of Paediatric and Adolescent Rheumatology, Royal Manchester Children’s Hospital, Manchester University Hospitals NHS Trust, Manchester, UK; 4 Sheffield Children’s Hospital and Sheffield Teaching Hospitals, Manchester, UK; 5 Bristol Royal Hospital for Children and Royal National Hospital for Rheumatic Diseases, Bath, UK; 6 Public Health Family Nurse, Family Nurse Partnership, Wallacetown Health Centre, NHS Tayside, Dundee, UK; 7 Paediatric Rheumatology European Society (PReS) Juvenile Dermatomyositis Working Party, Manchester, UK; 8 Paediatric Rheumatology, Newcastle University, Newcastle upon Tyne, UK; 9 Honorary Consultant Great North Children’s Hospital, Newcastle Hospitals NHS Foundation Trust, Newcastle upon Tyne, UK; 10 Chair Paediatric Global Musculoskeletal Task Force, Manchester, UK; 11 Consultant Paediatric Rheumatologist, Alder Hey Children’s NHS Foundation Trust, Liverpool, UK; 12 Honorary Clinical Senior Lecturer, University of Liverpool, Liverpool, UK

## Abstract

**Background:**

The importance of developmentally appropriate transitional care in young people with juvenile-onset rheumatic and musculoskeletal disease is well recognised. The Paediatric Rheumatology European Society (PReS) / European League Against Rheumatism (EULAR) Taskforce has developed international recommendations and standards for transitional care and a growing evidence base supports the positive benefits of such care. However, there is also evidence that universal implementation has yet to be realised. In 2020, against this background the COVID-19 pandemic arrived with significant impact on all our lives, young and old, patient, public and professional alike. The unfortunate reality of the pandemic with potential for unfavourable outcomes on healthcare provision during transition was acknowledged by the PReS working groups in a position statement to support healthcare professionals, young people and their caregivers.

**Aim:**

The aim of this review is to present the literature which provides the rationale for the recommendations in the PReS Position Statement.

**Summary:**

The following areas are specifically addressed: the prime importance of care coordination; the impact of the pandemic on the various aspects of the transition process; the importance of ensuring continuity of medication supply; the pros and cons of telemedicine with young people; ensuring meaningful involvement of young people in service development and the importance of core adolescent health practices such as routine developmental assessment psychosocial screening and appropriate parental involvement during transitional care.

## Introduction

Health transition is a triphasic process for young people (10–24 year olds). The first phase starts in early adolescence with a lengthy stage of preparation. This is followed by the second stage - the event of transfer, usually in late adolescence. The third phase following transfer is of variable length as young people gradually engage with the new adult service [1]. Effective implementation of health care during this time requires engagement from both paediatric and adult services with young people and their families / caregivers. Transitional care should prepare and support young people and their families / caregivers throughout this 10–15 year period to ensure optimal experience. Health transition addresses psychosocial, social, educational and vocational aspects of care in addition to the physical and medical issues relating to their underlying condition [1–3]. Promotion of the confidence of individual young people in managing their health condition has been reported to be associated with better transitional care outcomes [4]. Rheumatology services should therefore both support and nurture the development of health literacy and self-management skills to enable young people to gain such confidence. This takes time. Stollon et al. reported that half of health transition skills were acquired after the age of 18 years [5]. Throughout the transition process, healthcare provided should be developmentally appropriate [6]. Various toolkits are available to support work in this area [7, 8].

EULAR/PReS has provided professionals with standards and recommendations for the transitional care of young people with juvenile-onset Rheumatic and Musculoskeletal Diseases (jRMDs) [2]. Alongside such guidance, the evidence base to support transitional care has continued to grow [3] since the first objective evaluation of transitional care in juvenile idiopathic arthritis reporting positive outcomes over a decade ago [9]. However, the challenges of implementation remain. A Single Hub and Access point for Paediatric Rheumatology in Europe (SHARE) survey reported insufficient transition management planning [10]. A survey of adult rheumatologists in the USA reported significant unmet needs [11] and reports of country specific challenges further highlight the importance of considering the health systems within which rheumatology services are delivered and within which young people and their families have to learn to navigate [12].

### Impact of COVID-19 pandemic on young people

Although less at risk of the serious complications of the virus [13], the pandemic has had major impact on the psychological, social and vocational development of young people [14–18].

Social development is core to adolescence and social distancing strategies have had a major impact on peer/social support systems [14]. Social distancing has also contributed to anxiety and uncertainty [15] Young people may be stressed by the fear of exposure and submit themselves to self-quarantine even when not necessary. Some young people may also not have the emotional nor psychosocial resources to cope with the enormity of changes in daily life which the Covid-19 pandemic has brought [15–17].

In particular, school closure, social distancing and anxiety over health and threats to family employment/income have increased the risks of social isolation, emotional distress and/or mental ill health for young people [16, 17]. Finally, a significant number of the global population of young people are excluded from remote access to health and education due to lack of internet access, low levels of digital literacy or poverty [19]. Other challenges include, but are not limited to, learning disability, auditory or visual needs or language barriers.

### Impact of pandemic on health care

The impact of the pandemic on health services has been enormous [20–24]. From the young person and family perspective there is anxiety of coming into a hospital where there are covid infected patients although this is less so in paediatric hospital settings. Physical distancing requirements have led to less frequent face-to-face appointments and delay of investigations. There has been a move towards remote clinics, which brings additional challenges for YP, their families and health care providers. Furthermore, whereas young people can still be accompanied in hospital whether in face-to-face clinics or as inpatients in paediatric settings, they may not be able to be accompanied in adult clinics and wards.

The wearing of masks makes non-verbal communication (such as providing reassuring smiles) to the anxious young person as well as making it more challenging to hear the less articulate, quiet and/or reticent teenager.

### Background to the PReS position statement

Concern regarding the impact of change in healthcare services due to Covid-19, particularly affecting transition and transfer to adult services, was raised by the parent representative of the Juvenile Dermatomyositis (JDM) PReS Working Group and discussed within a JDM working party core group meeting. A decision was made to write an evidence-informed position statement to highlight this issue and help address the needs of young people with JDM transitioning to adult services during the Covid pandemic. A draft position statement was written (LMcC) and ratified by the core group of the JDM working party. When presented at the PReS JDM working party meeting during the PReS Conference (September 2020) there was a request from other Working Parties to make the transition statement generic for jRMDs. With the help of experts in adolescent and young adult (AYA) health [JMcD, RT, HF] and the JDM working party parent representative (JS) the draft statement was further modified. The refined position statement was submitted for review to the PReS Clinical Affairs group and Working Party Chairs. Following minor changes and approval by the PReS Council and Executive, the position statement was published on the PReS website [25].

The aim of the position statement was to define best practice during transition and transfer to adult care for AYA with jRMDs, and address the challenges of the necessary switch to less face-to-face and more remote contacts with YP and their families.

## PReS position statement

The position statement details 6 recommendations and signposts to useful resources (see Table 1).

**Table 1:** PRES Position Statement for transitional care during the covid-19 pandemic for young people with juvenile onset rheumatic and musculoskeletal diseases - abbreviated [25]

1. Paediatric and adult rheumatology teams should make every effort to put a coordinated transition process in place for young people.	
2. Paediatric and adult rheumatology teams should be aware of the potential impact of Covid-19 on transition processes and engage members of the multidisciplinary team to support young people and their families / caregivers through this process.	
3. During transfer from paediatric to adult services, processes should be in place to ensure that there is no disruption to supply of medication or other medical care that a young person receives.	
4. Consideration should be given to expansion of formal telemedicine protocols to help address continuity of care during covid-19 where possible.	
5. YP should be meaningfully included in discussions regarding service developments and use of telemedicine to aid transition processes.	
6. During the Covid-19 pandemic, general considerations regarding transition should be taken into account including: • Routine psychosocial screening. • Individualised transition planning which acknowledges impact of the pandemic. • Disease and symptom control including fatigue.	

These recommendations will be discussed further in detail below.

### Importance of coordination

Coordination is vital at every stage of transition - from early adolescence to young adulthood following transfer to adult hood and clearly stated in guidance to date [25, 26]. The pandemic has potentially impacted on this aspect of transitional care due to the impact on health service delivery [20–24] in addition to the impact on the vocational and social care agencies with whom health care teams need to coordinate care with, namely schools/colleges/universities and organisations providing social support for young people, so many of which closed during lock down. For example, one of the proposed beneficial features associated with positive transitional care outcomes is meeting with the adult team prior to transfer [26]. In an international Delphi study a trusting relationship with the adult care provider was felt to be essential and very important for successful transition by 86% of participants [27]. However, even before the pandemic, this can be a challenge in view of coordinating appointments when the adult rheumatologist can be physically present. For some adult rheumatology professionals it is impossible in view of geographical distance. An opportunity of the pandemic is the exponential growth of the use of video consultations when face-to-face consultations are not possible. Virtual consultations enable attendance of members from both paediatric and adult rheumatology team as well as other professionals in the context of multisystem diseases such as Systemic Lupus Erythematosus (SLE) thereby facilitating coordination of care particularly in the peri-transfer period.

There are various models of such “combined clinics” and evidence is still limited as to which model is best. It is important to note that some authors have reported limitations of such clinics [28]. Irrespective of model, it remains important that these clinics remain developmentally appropriate whether face-to-face or virtual e.g. is there time for the young person to be seen independently of the caregivers? How is privacy and confidentiality assured? Even prior to the pandemic, Jensen et al. reported that half of young people (16–23 year olds) were not seeing health professionals independently for at least part of the visit [29].

.

There is evidence to support the concept of a young adult clinic for 16–25 year olds as having better outcomes than the more traditional combined clinic with adult and paediatric rheumatology teams in the same consultation [30]. Reduced morbidity in young people with renal disease has similarly been reported with such models [31]. Young Adult Clinics also provide continuity at a time of many other transitions whether that be educational, living independently, entry into the workforce and furthermore acknowledges that transition continues after transfer. Research into this third phase is limited with only 14 of 71 studies in a systematic review focused on this phase [32]. The implementation of young adult clinics is both developmentally appropriate as well as enabling further research involving this age group.

### Importance of a multidisciplinary team (MDT)

As well as impacting the health care provision for young people, the pandemic has also affected multidisciplinary team working with social distancing restrictions limiting face-to-face meetings and the number of professionals physically present in clinic.

Successful engagement of YP in transitional care requires a team-based approach [33]. Team climate is used to describe shared perceptions of organisational policies and includes vision, commitment to excellence and innovation [34]. Change in the team climate has been reported to predict the quality of transitional care delivery [35] and therefore re-deployment and staff illness during the pandemic may have negatively affected the team climate accordingly. Attention is therefore required for teams to reflect on how the pandemic has impacted team working and address any negative impacts. One of the EULAR recommendations [2] is to have a written, agreed and regularly updated transition policy. A review of such written policies is recommended in the light of COVID restrictions. However, it should be noted that such written policies are not yet universal with only 27% of European paediatric centres reporting using them [36].

### Ensuring continuity of medication supply

Even in the absence of a pandemic, the transfer to adult services can be a vulnerable time for adherence particularly when young people have to start paying for their prescriptions as happens in some parts of the UK. Learning how to take responsibility for their own medication including the organisation of repeat prescriptions (“refills“) are core transitional readiness skills for all young people. The pandemic significantly impacted accessibility to family doctors and pharmacy services which potentially hindered such skills training although those services offering online ordering of repeat prescriptions were more enabling of young people for the acquisition of such skills. Learning to navigate the health service including pharmacy related aspects of self-care during the ever changing landscape during the pandemic was a particular challenging aspect of transitional care for young people and the health professionals teaching them alike.

Adherence has warranted particular attention during the pandemic as young people in remission may feel they don’t want to risk infection due to immunosuppression and therefore decide on their own volition to stop their biologic or Disease-Modifying anti-rheumatic drug (DMARD). It is important to revisit understanding of the benefits and risks of medication and the need to avoid unplanned admissions due to uncontrolled disease. For some in the peritransfer period, this would have meant their first ever admission to an adult ward where visiting of caregivers was severely restricted. High dose steroid rescue therapy for uncontrolled disease was often associated with a period of shielding with all the implications to social and vocational development and adolescent mental health [refs]. Finally this is particularly important in the light of recent reports of uncontrolled disease being a risk factor for COVID − 19 mortality in adults with rheumatic diseases [37]. Although this risk has not been not reported in adolescents, high dose steroid rescue therapy for uncontrolled disease was often associated with a period of shielding with the morbidity associated with the impact of this on social and vocational development and adolescent mental health [16].

### Expansion of formal telemedicine protocols

Adolescence is a time when young people start learning the skills to manage their own health, including talking with health professionals. This can be challenging enough for some in face-to-face consultations never mind virtual consultations particularly if the patient-professional relationship is not yet well established. Therefore, a face-to-face consultation, with the appropriate social distancing, personal protective equipment and hygiene control, may be preferable for young people whether they be new to a paediatric setting or transferring to a new team in adult services.

The pandemic has brought opportunities particularly with regards to the use of digital technologies in healthcare. Technology can be used to an advantage in this age group as factors that make technology use attractive are amplified in adolescence. Indeed, adolescents interact with technology more than any other age group [38]. Surveys indicate that young people view technology as having a positive or neutral effect on their social and emotional well-being [38].

One of the predictors of positive outcomes of transitional care has been reported to meeting the adult provider prior to transfer [4]. Prior to the pandemic, this was often considered to be impractical for many young people particularly if the adult service was geographcially distant. The expansion of telemedicine protocols however has enabled this to happen with joint virtual meetings with young people, their parent/caregiver, and both paediatric and adult team members for which toolkits are now available [39]. When access to telemedicine is limited, joint conference calls is another useful approach.

There is growing evidence that telemedicine is feasible for providing care to youth for a variety of health concerns [40], including group work [41]. However ‘one size does not fit all’ and although YP may view technology positively, this may not be true with respect to use in their education nor health care. Steps should be taken to ensure confidentiality is maintained such as ensuring the young person is in a private location if available, and /or use of chat function or headsets as needed [42]. Professionals should also not assume that all YP prefer digital communication. Development of body image is integral to adolescent development and may impact on use of videos in virtual consultation. This may be another reason for YP preferring either face to face or phone consultations. Scheduling of such consultations can be challenging too. For example, if a virtual appointment is during school/college hours, there may be lack of confidential space in the educational environment for the young person and similarly in the parental work place. It is therefore important to give young people the choice of format when at all feasible [42].

Thought should be given as to when physical examination is required. Face-to-face consultation may be preferable particularly if young people are still growing. Unlike younger children, parents don’t always see young people undressing so may not be aware of development of abnormalities. Physical examination is also a key opportunity to discuss puberty, growth, and their bodily concerns with young people. Even simple things like obtaining the weight of a young person to calculate drug dosages can be a challenge if families do not have access to scales!

Learning to navigate the health system is another core transitional care skill made so much more challenging by the pandemic when we have seen the system change and change again as we rapidly readjust to life in the time of a pandemic. This is a key area for health professionals to pay attention to during consultations and ensure that young people know how to navigate the current health system i.e. know who to go to for what and when and how. Such training now will need to address how young people navigate virtual as well as face-to-face consultations.

The distribution of electronic questionnaires and checklists has become easier with the increased use of telemedicine and digital communication between families and rheumatology teams. A number of resources have recently been developed to support healthcare professionals, YP and families in telemedicine, both generically [43, 44] and specific to paediatric rheumatology / musculoskeletal disease [45, 46].

There is huge potential for telemedicine beyond the pandemic to facilitate access to health care in settings where there is little or no specialist services available locally. However significant challenges need to be addressed before virtual consultations are adopted within routine clinical practice particularly with respect to confidentiality, quality of care and digital poverty [47].

### Including young people in service developments and use of telemedicine to aid transition processes

It is important to remember that ‘no size fits all’ as highlighted above [47–49] and examples of some of the pros and cons of telemedicine in transitional care are summarised in Table 2.

**Table 2:** Pros and cons of the use of telemedicine with respect to young people

Pros	Cons
Facilitate multidisciplinary involvement (including hospital as well as community based) especially if geographically distant	It is not the preference of all young people and may not be suitable for those with language barriers, auditory or visual needs or learning difficulties
Facilitate involvement of both paediatric and adult teams especially if geographically distant	Challenges in assuring confidentiality for the young person
Young people are familiar with the technology	More challenging to establish new relationships between health professionals and young people e.g. more difficult to pick up on nonverbal cues including between young person and the accompanying adult etc
Reduces the need for travel in both individual and group work	Limited access to private space
Distribution of questionnaires, screening tools and transition checklists prior to appointment	Limited access to technology
Reduces cost for families (avoiding travel / time off work)	Dislike of video use when young person has body image issues
	Limitation for physical examination (including pubertal assessment) and loss of opportunity to discuss bodily changes
	Challenges of scheduling with respect to educational/work commitments of young person and parental working patterns
	Safe-guarding issues of virtual examination and the receiving, capturing, storing and the use of images for clinical purposes
If used to preface physical clinics eg to take initial history etc. can reduce appointment time	Limitation for accurate measurement of height and weight
Keeping families connected during hospital stays	An additional skill for young people to learn

YP therefore need to be involved in discussions regarding service developments in the COVID 19-era particularly with respect to the use of telemedicine. Learning to navigate virtual as well as face-to face consultation is an additional skill for them to learn. Furthermore young people most in need of healthcare may be the most ‘digitally deprived’ as a result of poverty whether that be in terms of access to technology or private space to use that technology confidentially in; the drive to increased use of digital technologies may merely serve to increase health inequalities.

Involving YP from a range of socioeconomic backgrounds in service development has always been very important and was largely done face to face prior to the pandemic. Such involvement can now be readily facilitated by digital technologies and there are excellent resources to support professionals to work with YP virtually when face to face meetings are not possible due to social distancing restrictions [50].

### General considerations regarding transition

The Covid pandemic and the resulting media coverage has provided valuable opportunities to discuss particular aspects of health with young people including viruses, infection risk, role of vaccination etc. As in all adolescent health care, routine psychosocial screening with respect to emotional well-being, sleep, fatigue and exercise is integral to all consultations [43, 50] and even more important during a pandemic as all themes encompassed by such tools have been significantly impacted (Table 3). Tools such as HEEADDSSSS (Home, Education, Exercise, Activities, Diet, Drugs, Sleep, Safety, Suicide, Social Media [50, 51] or THRxEADS (Transition, Home, Treatment, Education/Exercise, Activities, Diet/Drugs, Sleep, Safety, Suicide, Social Media) [52] can easily be incorporated into consultations, face to face or virtual with young people .

**Table 3:** Impact of Covid 19 pandemic on areas of HEEADSSSS screening

	Impact of COVID19 pandemic on areas of young people’s lives
Home	Being at home has a very different impact depending on their circumstances e.g. Parental disharmony due to lockdown, unemployment, financial difficulties; limited space for studying or confidential conversations; exposure to violence – child abuse or domestic violence
Exercise	Limited opportunities to exercise during lockdown.
Education/work	Significantly impacted by the pandemic with disruption of exams and important rites of passage e.g. end of school proms; lack of face to face work experience opportunities; difficulties visualizing a future; loss of work opportunities in retail and hospitality industries.
Activities	Limited opportunities for hobbies, leisure activities, interaction with friends/peers; sudden removal of support networks via school, work etc.
Diet	Food poverty; weight gain due to lack of exercise and/or comfort eating.
Drugs	Increased use of drugs and alcohol due to lower mood.
Sleep	Loss of routine; resulting fatigue; lack of exercise during day; increased time online.
Safety	Increased time online.
Suicide/mood	Depressive and anxiety symptoms; worsening of existing mental health difficulties.
Sexual health	Difficulties accessing confidential sexual health advice.

HEADS and THREADS are useful strategies to both engage young people and find out about their lives but also to identify risk and protective factors which then assist in formulating future interventions whether that be to maximize adherence or how to orientate information giving or motivate. Questioning should be interactive and not interrogative, always leaving time for young people to answer. Active listening skills [52] and being comfortable with silence are key skills for rheumatology professionals irrespective of the format of the consultation. It is important to give warning before asking the more sensitive questions such as related to sexual or mental health. Healthcare professionals should always explain why they are asking such questions and remember that young people have the right not to answer, just like the rest of us! Unmet needs with respect to mental health provision as well as expertise in rheumatology services have been reported prior to the pandemic [53–56]. There remain potential unmet training needs with respect to effective communication with a discrepancy with what young people say is covered in such interviews and what the professional reports [57]. In view of the impact of the pandemic on the mental health of young people generally [16, 58], these needs are likely to have increased and compounded further by the negative impact on third sector providers of youth mental health support services. Training in adolescent health with particular attention to communication skills with digital technology remains an important component of developmentally appropriate transitional care [2, 59].

Practitioners should be mindful that jRMDs impact on the individual biopsychosocial development of YP as well as how that development influences jRMDs and therapy. Routine developmental assessment remains important as with any adolescent whether it be a face-to-face or virtual consultation. Individualised transition plans should be developed with active involvement of the young person so that they feel listened to and understood as an individual in their own right. Assessment of transition and transfer readiness using such plans are integral to every clinical encounter for young person.

Appropriate parental involvement during transitional care has also been reported to be associated with positive transitional care outcomes [4]. Parents of young people with long term health conditions report significant needs during this period [60]. Individualised transitional care plans, like those used with young people are also available to use with parents [61].

Every attempt should be made to keep the young person as well as possible and avoid any unplanned inpatient admissions particularly during the peri-transfer period. The first ever admission (emergency / routine) in an adult hospital during the peri-transfer period can cause unease at any time, potentially exacerbated by necessary processes during the Covid-19 pandemic. Preparation for this is important. The use of a patient held care plan may be helpful and could include considerations for blood tests or procedures to help alleviate anxiety [62].

## Conclusions

The COVID-19 pandemic has had a major impact on health care provision for all ages and the full impact on the lengthy process of transition has yet to be realised. The PReS Position Statement aims to raise awareness of the pandemic’s impact on YP with jRMD, the impact on various aspects of transition and the ‘pros and cons’ of telemedicine. Routine practices in adolescent health remain core to clinical practice i.e. psychosocial screening and developmental assessment as these effectively inform the practitioner of both risk and protective factors as well as how to effectively engage the young person. Further research is needed to determine the impact of the COVID pandemic on team working, use of telemedicine and the effective engagement of young people in their care. Further work is needed to define the best model to ensure developmentally appropriate rheumatology care during adolescence and young adulthood with particular attention to the less researched third phase of transition in adult services.

## Data Availability

Not applicable.

## Abbreviations

AYA: Adolescent and young adult
DMARD: Disease-Modifying Anti-Rheumatic Drug
EULAR: European League Against Rheumatism
HEEADDSSSS: Home, Education, Exercise, Activities, Diet, Drugs, Sleep, Safety, Suicide, Social Media
JDM: Juvenile Dermatomyositis
jRMDs: Juvenile-onset Rheumatic and Musculoskeletal Diseases
PReS: Paediatric Rheumatology European Society
SHARE: Single Hub and Access point for Paediatric Rheumatology in Europe
SLE: Systemic Lupus Erythematosus
UK: United Kingdom
THRxEEADDSSSS: Transition, Home, Treatment, Education, Exercise, Activities, Diet, Drugs, Sleep, Safety, Suicide, Social Media
YP: Young People
